# Physicochemical, Functional, and In Vitro Fermentation Characteristics of Buckwheat Bran Dietary Fiber Modified by Enzymatic Extrusion

**DOI:** 10.3390/foods14081300

**Published:** 2025-04-09

**Authors:** Tingting Bu, Yue Yu, Xiao Kong, Weicheng Wu, Zhiguo Zhang, Weiwei Hu, Komarova Natallia, Ming Cai, Kai Yang, Peilong Sun

**Affiliations:** 1 College of Food Science and Technology, Zhejiang University of Technology, Hangzhou 310014, China; butingting513@163.com (T.B.); 221122260071@zjut.edu.cn (Y.Y.); kongx0205@163.com (X.K.); caiming@zjut.edu.cn (M.C.); 2 Moganshan Institute ZJUT, Deqing 313200, China; 3 Food Science Institute, Zhejiang Academy of Agricultural Sciences, Hangzhou 310021, China; wuwc@zaas.ac.cn (W.W.); zhangkii@126.com (Z.Z.); huww@zaas.ac.cn (W.H.); 4 Scientific-Practical Center for Foodstuffs, National Academy of Sciences of Belarus, 220037 Minsk, Belarus; knv@belproduct.com

**Keywords:** buckwheat bran, dietary fiber, enzymatic extrusion, physicochemical properties, gut microbiota

## Abstract

The effects of cellulase–xylanase synergistic treatment combined with twin-screw extrusion on the physicochemical, functional, and in vitro fermentation characteristics of buckwheat bran dietary fiber (BBDF) were investigated. Compared to single enzymatic hydrolysis, the synergetic modification was more effective in promoting the soluble DF (SDF) ratio (increased from 10.68% to 32.67%), functional properties, and prebiotic activities of BBDF and decreasing the insoluble DF (IDF) content. Under 0.6% (*w*/*w*) cellulase and xylanase with mild extrusion conditions (40–80 °C), the modified BBDF exhibited the highest capacities for glucose and cholesterol adsorption. FTIR and XRD experiments indicated that the enzymatic extrusion destroyed the intermolecular interactions of BBDF. Furthermore, enzymatically extruded BBDFs showed 2.2-fold higher short-chain fatty acid (SCFA) yields during in vitro fecal fermentation (total SCFAs: 87.8 mM vs. 40.0 mM in control), with butyrate production reaching 2.5 mM (+76.3%), among which the mildly extruded BBDFs exhibited superior prebiotic effects.

## 1. Introduction

Buckwheat is a pseudocereal crop belonging to the genus *Fagopyrum* of the family *Polygonaceae* [1]. Bran is a byproduct of buckwheat processing with a variety of natural compounds, such as dietary fiber (DF), protein, flavonoids, and phenolics. DF makes up about 40% of buckwheat bran (BB), in which insoluble DF (IDF) accounts for the majority, which causes the poor texture and palatability of BB [2]. Compared with IDF, soluble DF (SDF) has superior functional characteristics (such as water holding capacity (WHC), oil holding capacity (OHC), and rheological property) and prebiotic effects [3]. Therefore, finding suitable modification methods to improve the functional properties of BBDF can improve the utilization of BB.

The modification of DF mainly includes chemical, enzymatic, and physical methods [4]. Screw extrusion is widely used as a means of physical modification with high efficiency, continuity, and flexibility. Shear forces and heating are introduced into the DF by the rotating twin screws, which melt and further expand the materials at the exit of the die due to an instantaneous change in pressure [5]. The thermomechanical processes during screw extrusion result in physical (e.g., water binding and swelling) and chemical (e.g., glycosidic bond breaking and fiber solubilization) modifications of DF in the feedstock [6]. Nowadays, an integrated approach with multiple modification methods can meet diversified needs and make the modification more thorough under milder processing conditions [4]. Compared with single twin-screw extrusion treatment, enzymatic extrusion, which combines controlled cellulase–xylanase hydrolysis and high-shearing extrusion, can achieve the shaping and biotransformation of raw materials simultaneously and has lower energy consumption [7]. For example, Song et al. revealed that combined cellulase–extrusion-treated bamboo shoot DF had a significantly increased SDF content and improved functional properties [8]. Xue et al. found that *Lentinula edodes* DF modified by twin-screw extrusion had higher glucose and bile acid adsorption capacities [9]. An in vitro fecal fermentation study found that eight widely consumed whole grains treated with extrusion had an increased total dietary fiber ratio, boosted microbial diversity, and promoted prebiotic activity [10].

Yet, most previous studies on DF modification have focused on dividing extrusion and enzymatic hydrolysis into two individual steps, which has limited the efficiency of further expanded production in industry [7]. Although some research has demonstrated the synergistic effect of α-amylase reactive extrusion, which is a one-step enzymatic extrusion approach, on the modification of starch and protein [11], a comprehensive analysis of the enzymatic extrusion of DF has not been reported. Moreover, during the one-step enzymatic extrusion process, the optimum temperature of cellulase and xylanase (about 50–70 °C) is usually lower than the temperature of screw extrusion (varying from 70 to 170 °C); thus, the extrusion temperature is a vital factor influencing the effectiveness of enzyme activity. Stepwise extrusion not only entails higher energy expenditure but also demonstrates operational inefficiency due to discontinuous phase transitions. Synchronizing the extrusion process with enzymatic catalysis through temperature optimization (within the enzyme’s activity range) achieves three synergistic effects: (1) process simplification by eliminating intermediate cooling/reheating steps; (2) energy conservation through thermal synergy between mechanical shear and biochemical reactions; and (3) enhanced catalytic efficiency via induced conformational changes in enzymes during extrusion, which promote the formation of enzyme/substrate complexes [12].

To address the abovementioned issues, this study investigated the effects of twin-screw extrusion (low or high temperatures) combined with cellulase–xylanase hydrolysis on the modification of BBDF. Specifically, BB was firstly premixed with different concentrations of cellulase and xylanase and further transported into the twin-screw extruder for the synchronized enzyme extrusion process. The physicochemical and functional properties of the modified BBDF were characterized. The pros and cons of the modified BBDF on SCFAs and gut microbiota composition were assessed through an in vitro fecal fermentation simulation. This exploration aims to give insights into the development of buckwheat-bran-based functional foods.

## 2. Materials and Methods

### 2.1. Materials and Reagents

Dried buckwheat bran (BB) powder was obtained from Xichang Zhengzhong Food Co., Ltd. (Chengdu, China). Heat-stable α-amylase (4000 U/g), glucoamylase (100 U/mg), cellulase (11,000 U/g), xylanase (280 U/mg), and alkaline protease (200 U/mg) were purchased from Yuanye Biological Technology (Shanghai, China). Acetic acid, propionic acid, butyric acid, isobutyric acid, isovaleric acid, and valeric acid were GC-grade and purchased from Aladdin Biochemical Technology Co., Ltd. (Shanghai, China). All other chemicals were of analytical grade and purchased from Sinopharm Chemical Reagent Co., Ltd. (Shanghai, China).

### 2.2. Enzymatic Extrusion of BB

The enzymatic extrusion followed the method of Guan et al. with slight alterations [13]. First, BB was mixed with the enzyme mixture (cellulase–xylanase = 1:3, *w*/*w*) at loadings of 0%, 0.2%, and 0.6% (*w*/*w*, enzyme/substrate). The mixture was adjusted to 30% moisture content by adding water and was then placed in zip-lock bags to equilibrate moisture at 4 °C. After equilibration for 12 h, the BB was passed through a twin-screw extruder (FMHE22-32, Fumarco, Changsha, China) at a feed speed of 10 kg/h and a screw speed of 150 rpm. The extruder barrel was regulated by seven heated barrel sections with individual temperature control, and in this test, two temperature conditions (low-temperature gradients of 40, 50, 60, 70, 80, 70, and 60 °C and high-temperature gradients of 80, 90, 100, 110, 120, 110, and 100 °C) were maintained separately. After extrusion, the BB was collected and dried in an oven at 50 °C until the moisture was below 13% (*w*/*w*) and further ground into powder and stored at 4 °C for analysis.

### 2.3. Preparation of BBDF

BBDF was extracted following the method of Wang et al. with slight alterations [14]. Briefly, the enzyme-extruded BB powder was soaked in 85% ethanol (1:10, *w*/*v*) for 3 h and filtered through gauze. The residue was extracted with distilled water (1:10, *w*/*v*), followed by treatment with thermostable α-amylase (1.5%, *v*/*v*, 3 h, 95 °C) and porcine pancreatic protease (0.5%, *w*/*v*, 3 h, 37 °C) and enzyme inactivation (15 min, 100 °C). The supernatant was separated from the insoluble residue by certification (5000× *g*, 10 min) and further precipitated with 95% ethanol (1:4, *v*/*v*). Then, the concentrate was rotary-evaporated to remove ethanol and freeze-dried to obtain BBDF.

### 2.4. Chemical Composition

The TDF, SDF, and IDF contents of control and different modified BBDF samples were determined using the method of Wang et al. [14]. The IDF composition was determined using lignin, cellulose, and hemicellulose kits (Edison Biotech Ltd., Zhenjiang, China) following the instructions. The Congo red test was conducted according to the method of Yang et al. to verify the trihelical structures of BBDF. The monosaccharide composition was analyzed by high-performance liquid chromatography (HPLC) [15]. Briefly, samples of 20 mg were decomposed with 2 M trifluoroacetic acid at 121 °C for 2 h and were then dried under nitrogen. The residue was subsequently dissolved in 1 mL of deionized water, filtered through a 0.22 μm membrane, packed into vials, and analyzed with high-performance anion-exchange chromatography (HPAEC) (ICS 5000+, Thermo Fisher Scientific, Waltham, MA, USA), equipped with a pulsed amperometric detector (PAD) and a Dionex™ CarboPac™ PA-20 column (3 × 150 mm, 10 μm). The molar ratio of the monosaccharides of BBDF samples was determined by comparing the retention times and peak areas of monosaccharide standards.

### 2.5. Particle Size

The control and different modified BBDF samples were dispersed in deionized water at 5 mg/mL. Then, the particle size distribution and Dx_90_ of the suspended samples were determined using a laser particle size analyzer (S3500, Microtrac, San Diego, CA, USA). The shading rate was controlled at 10–12% during the experiment.

### 2.6. Water Holding Capacity (WHC), Oil Holding Capacity (OHC), and Swelling Capacity (SC)

The WHC and OHC of control and modified BBDF samples were determined using the method of Liu et al. with some modifications [16]. In total, 0.5 g of each sample was weighed and mixed with distilled water (30 mL) or corn oil (10 g) in a centrifuge tube. After stirring for 1 h at 25 °C, the mixture was centrifuged for 15 min at 8000× *g*. The excess water or oil was removed and weighed. The WHC and OHC were calculated from the weight difference (g/g). The SC was determined following the method described by Geng et al. [17]. Each sample (0.1 g) was added to a graduated cylinder, and the original volume was recorded. Then, 5 mL of distilled water was added. The final volume of the swollen fiber was recorded after the hydration reaction for 1 h at 25 °C. The SC was expressed as the water absorption expansion volume per gram of the dry sample (mL/g).

### 2.7. Glucose and Cholesterol Absorption Capacity (GAC and CAC)

The GAC and CAC were determined using the method of Liu et al. with some modifications [18]. Briefly, control and modified BBDF samples (100 mg) were mixed with 50 mmol/L glucose solution (50 mL) and 10% (*w*/*v*) fresh egg yolk emulsion, respectively, and kept at 37 °C for 4 h. The mixture was centrifuged at 5000× *g* for 10 min at 25 °C to separate the supernatant. The glucose content and the cholesterol content in the supernatant were determined using the phenol–sulfuric acid method and o-phthalaldehyde method, respectively. The GAC (g/g) and CAC (g/g) were calculated as follows:

GAC and CAC = (W_O_ − W_e_)/W_S_

where W_O_ is the original glucose and cholesterol content (mg/mL), W_e_ is the glucose and cholesterol content once the adsorption reached equilibrium (mg/mL), and W_S_ is the weight of BBDF samples (mg).

### 2.8. Rheological Properties

The apparent viscosity and shear stress of control and modified BBDF samples were measured using a rheometer (MCR 302, Anton Paar, Graz, Austria). Each sample (3 g) was mixed with distilled water (3 mL), then placed on a PP50 parallel plate (50 mm diameter, 1 mm gap) and tested at 25 °C with shear rates ranging from 0.1 s^−1^ to 1000 s^−1^. The linear viscoelastic region was determined by stress scanning at 1 Pa stress at 25 °C. Subsequently, a dynamic frequency scan was conducted between 0.5 and 130 rad/s to record the storage modulus (G′) and the loss modulus (G″).

### 2.9. Fourier-Transform Infrared Spectroscopy (FT-IR)

The FT-IR spectra of control and modified BBDF samples were recorded using a Nicolet FT-IR spectrograph (Thermo Fisher Scientific, Waltham, MA, USA). The ground samples were mixed with KBr and then compressed into a 1 mm thick tablet. The spectral range was 4000–400 cm^−1^.

### 2.10. X-Ray Diffraction (XRD)

The XRD of control and different modified BBDF samples was examined by an Empyrean X-ray diffractometer (Malvern Panalytical, Malvern, UK) using Cu Kα radiation (1.5406 Å) generated at 40 kV and 40 mA. The powdered BBDF samples were scanned from 5° to 50° (2θ) at 2°/min, and the crystallinity was calculated using Jade software (version 6.0, Materials Data, Livermore, CA, USA).

### 2.11. Thermo-Gravimetric Analysis (TGA)

The thermal properties of control and different modified BBDF samples were analyzed by thermo-gravimetric analysis (TGA, SDTQ-600, TA Instruments, New Castle, DE, USA). Each sample of ~8 mg was heated from 25 °C to 700 °C at a rate of 20 °C/min. Nitrogen (99.99%) was used as the inert gas for the atmosphere at a flow rate of 50 mL/min.

### 2.12. Scanning Electron Microscopy (SEM)

The morphological characteristics of control and different modified BBDF samples were observed by scanning electron microscopy (SEM, S3400N, HITACHI, Naka, Japan). Freeze-dried samples were placed on double-sided carbon tape and coated with a thin layer of gold. Representative microscope images were collected at 2500× magnification.

### 2.13. In Vitro Gastrointestinal Digestion

The in vitro simulated gastrointestinal digestion method was used, including simulated gastric fluid (SGF) and intestinal fluid (SIF) prepared using the INFOGEST model with slight modifications [19]. For simulated gastric digestion, 8 mL SGF and 8 mL BBDF solution (200 mg/mL) were mixed and incubated in a 37 °C water bath shaker, and the pH of the mixture was adjusted to 3.0 with 6 M HCl. After 2 h of incubation, the pepsin was inactivated with 1 M NaHCO_3_. Then, 16 mL SIF was subsequently added for intestinal digestion. The pH of the mixed solution was adjusted to 7.0 with 6 M NaOH. The mixture was incubated at 37 °C for 4 h and terminated by heating at 90 °C for 10 min. The resulting samples were stored at 4 °C for in vitro fermentation experiments.

### 2.14. In Vitro Fecal Fermentation and Analysis

#### 2.14.1. Donor Recruitment and Fecal Sample Processing

Five males and five females aged 20–25 years old, with a body mass index ranging from 19 to 23 kg/m^2^, were enrolled in this study. All participants had no history of intestinal diseases, maintained their regular dietary habits, and had not consumed antibiotics or prebiotics within the past three months [20]. All volunteers provided informed and written informed consent, and this study was approved by the Ethics Committee of Zhejiang Academy of Agricultural Sciences (No. 202401047532).

Feces samples were taken from the 10 volunteers in the morning on the same day and were immediately collected in sterilized plastic tubes. Each gathered feces sample of 0.8 g was placed in an automatic feces machine (Halo Biotechnology Co., Ltd., Suzhou, China) with 8 mL of PBS buffer (0.1 M, pH 7.0) and vortexed evenly. The above operations were completed within 2 h.

#### 2.14.2. Medium Composition

The YCFA basal medium was prepared by dissolving 0.45 g KH_2_PO_4_, 0.45 g K_2_HPO_4_, 0.05 g NaCl, 0.064 g CaCl_2_·2H_2_O, 0.09 g MgSO_4_·7H_2_O, 2.5 g yeast extract, 10 g tryptone, 2 mL hemin solution, 1 g L-cysteine, and 200 μL Vitamin K_1_ in 1 L deionized water. For the experiments, the blank group (NC) was 100 mL of YCFA medium, and the control and modified BBDF groups were 100 mL of YCFA medium with 0.8 g of different BBDF samples added. Then, 500 μL of fecal diluent was added to the blank and experimental groups and cultured in an anaerobic incubator at 37 °C. A peristaltic pump was used to dispense 5 mL of each prepared medium into vials under nitrogen conditions, and all vials were sterilized by autoclaving. Each sample of 1 mL was collected at 0 h and 24 h for relevant analysis.

#### 2.14.3. Gas Production and pH

At 0 h and 24 h, 1 mL of each fermentation broth was collected to measure the pH value using a pH meter (PHS-3C, Hangzhou Aolilong Instrument Co., Ltd., Hangzhou, China). The concentrations of CO_2_, H_2_, NH_3_, and H_2_S in the fermentation flask were quantified using a gas detector (Hangzhou Hailu Medical Technology Co., Ltd., Hangzhou, China).

#### 2.14.4. Short-Chain Fatty Acid (SCFA) Analysis

The quantification of SCFAs was conducted according to the method of Cai et al. with some modifications [21]. Each fermentation broth of 1 mL was mixed with 3 mL of ethyl acetate and sonicated on ice for 10 min; the organic phase was collected, and the extraction was repeated three times. The gathered organic phase was filtered through a 0.22 μm organic filter membrane and analyzed by a gas chromatograph (6890N, Agilent Technology, Santa Clara, CA, USA) fitted with a flame ionization detector and DB-FFAP column. The employed conditions were as follows: N_2_ of 25 mL/min, air flow rate of 300 mL/min, H_2_ flow rate of 30 mL/min, inlet temperature of 250 °C, and detector temperature of 280 °C. Temperature programming was conducted, with the initial temperature of the column set to 80 °C for 2 min, followed by heating at a rate of 6 °C/min to a final temperature of 180 °C, which was maintained for 4 min. A sample injection volume of 1 μL was considered.

#### 2.14.5. 16S rDNA Amplicon Sequencing of Gut Microbiota

After 24 h of fermentation, microbial DNA in the samples was extracted by the CTAB/SDS method and subsequently sequenced by Novogene Biotechnology (Beijing, China) [17]. High-throughput sequencing of the 16S rDNA V4 region was conducted using the Illumina Miseq platform. Microbiological complexity and intergroup differences were investigated using high-quality clean data based on operational taxonomic units and annotation results.

### 2.15. Statistical Analysis

Data are presented as the mean ± SD. Experimental data were analyzed by one-way ANOVA and the SPSS software (version 24, SPSS Inc., Chicago, IL, USA). The Shapiro–Wilk test was used to evaluate the normal distribution of data. The chi-square test was performed to compare the rates. The mean ± standard deviation of triplicate samples is presented. A *p* value < 0.05 was considered to represent statistically significant differences between the compared groups.

## 3. Results and Discussion

### 3.1. Chemical Composition of BBDF

The chemical composition of BBDF after enzymatic extrusion is shown in Table 1. The TDF and IDF contents in BBDF were significantly reduced after enzymatic extrusion, which was partly due to the high pressure and high shear forces of extrusion destroying the glycosidic bonds between IDF and converting it into small-molecule SDF [22]. Meanwhile, the cellulase and xylanase cleaved the cellulose and hemicellulose, which disrupted the bonding among cellulose, hemicellulose, and lignin [23]. Notably, the SDF contents of the high-temperature group were higher than those of the low-temperature groups in the absence of the enzyme mixture and at 0.2% enzyme mixture addition. Meanwhile, the SDF content of the L-0.6% group was significantly higher than that of the H-0.6% group. This result may be due to the high temperature and high shear forces, which played dominant roles in releasing SDF at a relatively low enzyme content. When the enzyme addition was increased, the low-temperature groups were more suitable for the activities of cellulase and xylanase [8]. These activities improved the efficiency of the enzyme during the treatment, which resulted in the release of more SDF. In terms of the IDF composition, cellulose and lignin were the main components of IDF (approximately 80%), with a low content of hemicellulose. Extrusion significantly decreased the content of cellulose and lignin, and the addition of cellulase–xylanase further broke the structure of IDF, leading to reduced contents of cellulose, hemicellulose, and lignin. Specifically, the L-0.6% group exhibited the lowest IDF composition, which indicates that the enzyme complex and extrusion could work synergistically at a low temperature, which aligns with the results of the IDF content.

The monosaccharide composition of the BBDF samples consisted of arabinose, glucose, galactose, and xylose. Among them, glucose accounted for 77.85–82.82% in different groups, which suggests that glucose is the main component of BBDF. Approximately 12% of arabinose, 5% of galactose, and 4% of xylose were detected, and their contents showed a decreasing trend after extrusion treatment. Zou et al. illustrated that extrusion treatment reduced the proportion of arabinose and xylose in IDF from sweet potato residue, which may be related to the degradation of arabinoxylan [24]. Moreover, the destruction of arabinose and galactose by low-temperature extrusion was more severe, and the difference further widened with the increase in enzyme addition. The difference between the monosaccharide compositions of BBDF samples may be due to the degradation of IDFs by cellulase and xylanase, as well as the high pressure and strong physical shear during extrusion [25].

**Figure 1:**
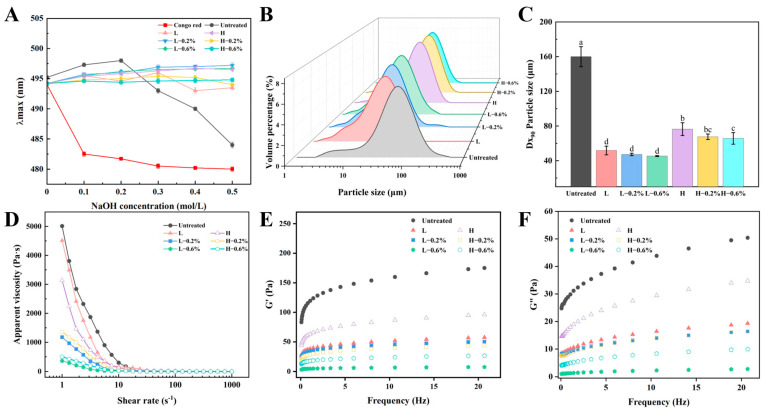
Structural and physical properties of enzymatic-extrusion-modified BBDF: the Congo red results (**A**), particle size distribution (**B**), Dx_90_ particle size (**C**), apparent viscosity (**D**), storage modulus (**E**), and loss modulus (**F**) of untreated and enzymatic-extrusion-modified BBDFs. L, low-temperature extrusion with a gradient of 40 °C, 50 °C, 60 °C, 70 °C, 80 °C, 70 °C, and 60 °C; H, high-temperature extrusion with a gradient of 80 °C, 90 °C, 100 °C, 110 °C, 120 °C, 110 °C, and 100 °C; 0.2% and 0.6%, the addition ratio (*w*/*w*) of the cellulase–xylanase mixture. a–d: BBDF (mean ± SD) under different processing conditions with differing letters indicating significant intergroup differences.

The Congo red experiment was also conducted to verify the triple-helical structure of modified BBDFs, and the results are shown in Figure 1A. The maximum absorption wavelengths of untreated BBDF were significantly decreased with increasing NaOH concentrations, indicating triple-helical structures in the BBDF. The modified BBDFs did not show significant changes in the maximum absorption wavelengths as the NaOH concentrations increased, indicating that the triple-helical structures of BBDF were destroyed after enzymatic extrusion. The exposure of the functional groups in the polysaccharide conformation can improve the functional properties and biological activity [15].

### 3.2. Particle Size of BBDF

The particle size of BBDF is a crucial factor that impacts its functional properties. The particle size distribution and Dx_90_ particle size of untreated and enzymatic-extrusion-modified BBDF samples are shown in Figure 1B,C. The Dx_90_ of BBDF was reduced from 160 μm to 45.4–76.4 μm after enzymatic extrusion treatments. All groups showed a single-peak distribution, and the distribution curves shifted to the left with narrower peak widths after processing, which could be attributed to the high pressure and high shear forces of extrusion disrupting the microstructure of the fiber and lowering the degree of aggregation of the DFs. These conditions led to the degradation of the cellulose and a reduction in particle size [26]. The particle sizes of the low-temperature group (45.4–51.8 μm) were significantly smaller than those of the high-temperature group (65.7–76.4 μm), likely due to the preservation of enzyme activity under milder thermal conditions. In addition, the particle size decreased slightly with the increase in enzyme addition, which suggests that enzymatic hydrolysis can further hydrolyze IDF to SDF with small molecules.

### 3.3. Rheological Properties of BBDF

As shown in Figure 1D, all BBDF samples showed similar flow patterns. The apparent viscosity of untreated and enzymatic-extrusion-modified BBDF samples decreased with the increase in the shear rate, which suggests that the BBDF suspension is a non-Newtonian fluid with shear-thinning behavior [27]. After extrusion treatment, the apparent viscosities of BBDF samples all showed a decreasing trend: untreated > high-temperature group > low-temperature group. The addition of the enzyme mixture further reduced the apparent viscosity. This result may be related to the particle sizes of the modified BBDFs being smaller than those of the unmodified BBDF. The smaller structure allows for better flowability, which may be a reason for the decrease in viscosity [28]. Moreover, the decreased proportion of viscous saccharides, such as arabinose and galactose, also led to a decrease in apparent viscosity [26]. Reduced viscosity was also found in the previously reported enzymatically homogenized DF from tomato pomace [29]. Extrusion and enzymatic hydrolysis jointly resulted in a reduction in the apparent viscosity of BBDF.

The G′ and G″ curves of untreated and modified BBDFs are shown in Figure 1E,F, in which G′ and G″ represent the solid-like and liquid-like characteristics of a viscoelastic material, respectively. As the oscillation frequencies increased, the G′ and G″ values increased steadily. The G’ and G″ curves of the low-temperature extrusion groups were lower than those of the high-temperature extrusion groups, and the G′ and G’’ values were further decreased with the addition of the enzyme mixture. This result suggests that the extrusion treatment reduced the viscoelasticity of BBDF to a certain extent, and the low-temperature treatment damaged the viscoelasticity more. The energy storage modulus (G′) was consistently higher than the loss modulus (G″) for all the samples in the experimental frequency range. Therefore, the elastic properties of BBDF were superior to the viscous properties, which resulted in weak gel properties [28].

### 3.4. WHC, OHC, and SC

**Figure 2:**
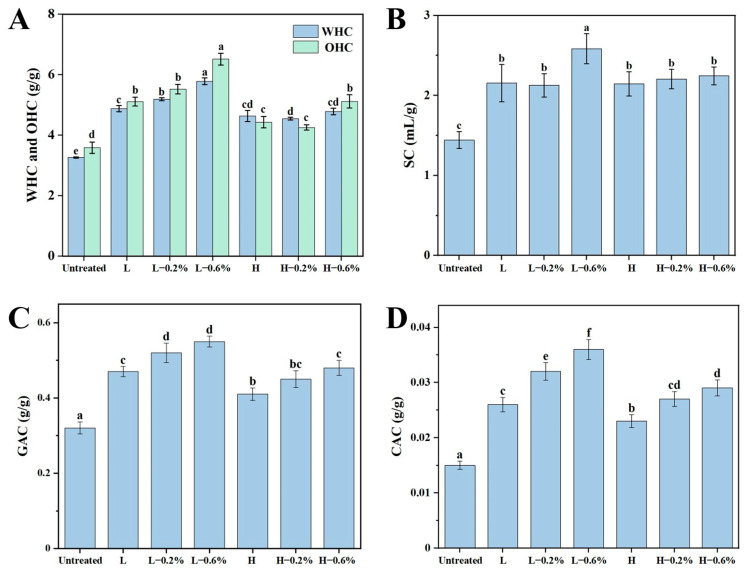
WHC and OHC (**A**), SC (**B**), GAC (**C**), and CAC (**D**) of untreated and enzymatic-extrusion-modified BBDFs. a–f: BBDF (mean ± SD) under different processing conditions with differing letters indicating significant intergroup differences.

The WHC, OHC, and SC responded to the adsorption and swelling properties of BBDF, which indicates the ability of BBDF to bind and swell with water/oil after absorption. As shown in Figure 2A,B, the WHC, OHC, and SC of the untreated and enzymatic-extrusion-modified BBDF samples were significantly increased after enzymatic extrusion treatment. Low-temperature extrusion enhanced the adsorption capacity of the samples more significantly than high-temperature extrusion. The difference between the two groups was further expanded after the addition of the enzyme, which is consistent with the results of the SDF content and particle size. The smaller average DF particle size was positively correlated to a higher binding capacity to water or oil [21,30]. This trend may be due to the enzymatic extrusion breaking glycosidic bonds within polysaccharide chains. This process reduced the particle size of the BBDF, increased the surface area, and exposed more hydrophilic/lipophilic groups, which improved the adsorption performance of the BBDF [31]. In terms of the SC, the enzymatic extrusion treatment increased the swelling capacity of DF, while the differences between the groups were not significant except for the L-0.6% group. Overall, enzymatic extrusion improved the water/oil adsorption properties and swelling characteristics of the BBDF, and the low-temperature extrusion groups had superior performance compared to the high-temperature extrusion groups.

### 3.5. GAC and CAC

The in vitro GAC and CAC of BBDF samples are presented in Figure 2C,D. The GAC and CAC are recognized as crucial indicators for evaluating the hypoglycemic and hypolipidemic potentials of DF [18]. Compared with the unmodified BBDF, all the modified BBDF samples exhibited an increased GAC and CAC. Moreover, the increase in the GAC and CAC after adding the enzyme mixture occurred in a dose-dependent manner. Among the modified BBDF groups, the low-temperature extrusion groups had a higher GAC and CAC than the high-temperature extrusion groups under the same enzyme mixture content. The reason for this may be the smaller particle sizes of low-temperature-extruded BBDFs, which extend the contact area with glucose and cholesterol and expose more functional groups (such as aldehydes and hydroxyl), thus enhancing the wrapping capacity for glucose and cholesterol molecules [32].

### 3.6. FT-IR

**Figure 3:**
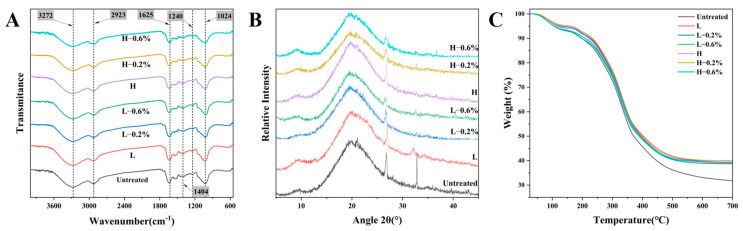
FT-IR spectra (**A**), XRD patterns (**B**), and TGA curves (**C**) of untreated and enzymatic-extrusion-modified BBDFs.

FT-IR enables the qualitative determination of differences in the chemical bonds and functional groups of BBDFs modified by enzymatic extrusion. As displayed in Figure 3A, the spectral patterns of all DFs were similar, while the intensity of the absorption peaks varied at characteristic wavenumbers. The intensity and width of the broad absorption peak near 3272 cm^−1^, which was attributed to the stretching vibration of the hydroxyl group, were affected by inter- and intramolecular hydrogen bonding [33]. The absorption peak at 2923 cm^−1^ was due to the stretching of the C–H vibrational bands of methyl and methylene groups in polysaccharides. The treated samples were redshifted from 3272 cm^−1^ to 3268 cm^−1^, and the intensity of the peak at 2923 cm^−1^ was weakened, which suggests that the hydrogen bonding in cellulose and hemicellulose was broken and the number of hydrophilic sites increased. The stretching peak at 1625 cm^−1^ corresponded to that of the benzene ring in lignin. The peaks at 1240 and 1024 cm^−1^ were C–O vibrations of methoxy in lignin and hemicellulose [34], and the intensities of these two peaks in BBDF were reduced after enzymatic extrusion, which implies intramolecular degradation or structural changes in cellulose and hemicellulose. Overall, the changes in the intensity of the characteristic absorption peaks of the modified BBDF indicated the degradation and destruction of the cellulose components.

### 3.7. XRD

The crystalline structure of DF is mainly caused by crystalline cellulose, and the non-crystalline structure is mainly caused by non-crystalline cellulose, hemicellulose, and lignin. As shown in Figure 3B, all BBDF samples exhibited a prominent primary diffraction peak at approximately 20°, along with secondary diffraction peaks at 26.8° and 32.8°, which are typical characteristics of the crystalline lattice of cellulose I [35]. After extrusion treatment, the intensity of the crystalline and amorphous peaks decreased, and the damage to the crystal structure was greater with high-temperature extrusion. After enzyme addition, the amorphous zone was further reduced or even disappeared. Liu et al. reported that cellulase treatment reduced the crystallinity of DF from rice bran, which was attributed to the action of cellulase on the β-1-4 glycosidic bond of cellulose; it ultimately degraded crystalline cellulose to a soluble cellulose degradation product [18]. Ma et al. (2023) reported that extrusion treatment reduced the crystallinity of DF extrusion in brown rice, which was related to the disruption of hydrogen bonding in cellulose [36]. Consistent with the results of this study, enzymatic hydrolysis, high pressure, and high shear forces disrupted the crystalline regions of BBDF, which weakened inter- and intramolecular hydrogen bonding forces and facilitated the release of amorphous components [3].

### 3.8. TGA

The thermal stability and structural properties of untreated and enzymatic-extrusion-modified BBDF samples were evaluated by TGA. As shown in Figure 3C, the weight of BBDF decreased slightly between 50 °C and 200 °C. The decrease in weight was mainly due to the water adsorbed by the hydrophilic groups of the fiber [30]. Specifically, the thermal degradation of cellulose and hemicellulose resulted in a rapid weight loss of BBDF between 200 °C and 400 °C, whereas weight loss at 400–600 °C was mainly due to carbonylation [37]. The results demonstrated that enzymatic extrusion improved the thermal stability of BBDF samples, although no significant differences were observed between treatment groups, indicating that the high pressure and high shear in the extrusion process had a greater effect on thermal stability, while the addition of the enzyme treatment insignificantly affected the thermal stability of BBDF. Previous studies have shown that extrusion can improve the thermal stability of BBSDF, which is consistent with the results of this study [27].

### 3.9. Morphological Structure

**Figure 4:**
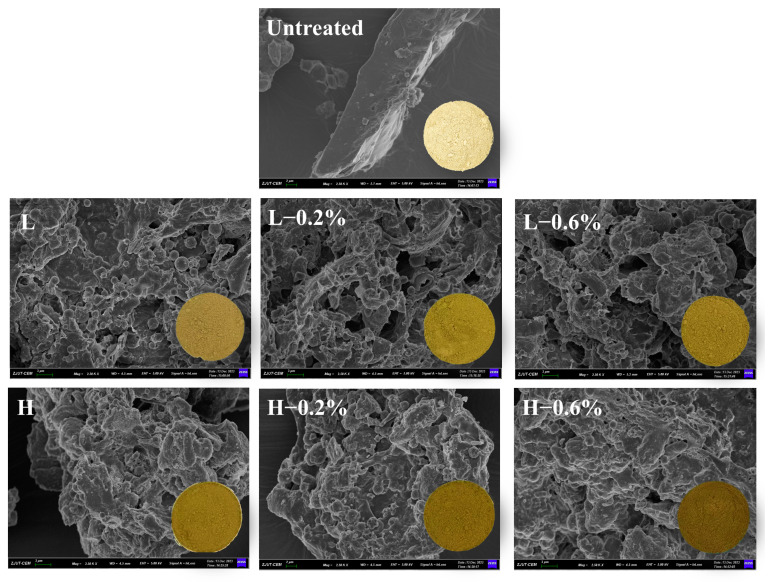
Appearance and SEM images (2500×) of untreated and enzymatic-extrusion-modified BBDFs.

The morphological structure of untreated and enzymatic-extrusion-modified BBDF samples is shown in Figure 4. The unmodified BBDF had a light-yellow color, and the surface structure of the unmodified BBDF was dense and smooth with no pores. After extrusion treatment, the surface became rough with grooves, folds, and a honeycomb structure, and the color turned dark and brown. The structural change might be due to the extrusion-induced breakage of cellulose and hemicellulose glycosidic bonds, which led to microstructural reorganization [38]. The honeycomb structures of the modified BBDF might lead to an increase in the WHC, OHC, and SC [29]. Notably, the microstructure of the low-temperature group was significantly looser with larger gaps and more porous after the addition of enzymes. This finding may be due to the low-temperature group being more suitable for the action of enzymes, which led to further structural disruption, the hydrolysis of cellulose and hemicellulose, and the generation of honeycomb structures [31].

### 3.10. Gas Production and pH of BBDF

**Figure 5:**
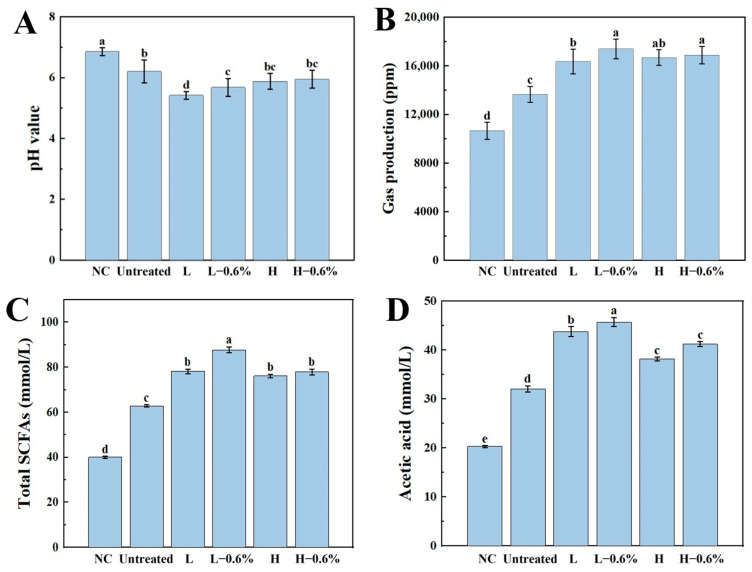

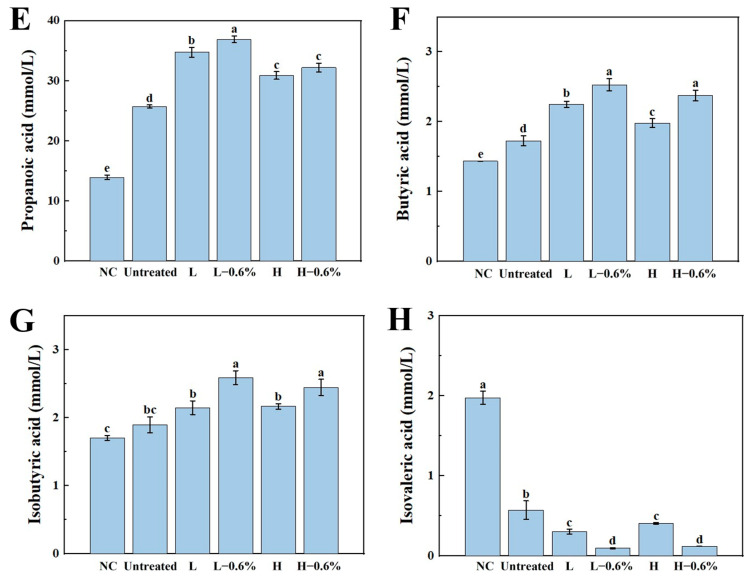
Effects of enzymatic-extrusion-modified BBDF on the gas production (**A**), pH value (**B**), and SCFA concentrations (**C**–**H**) after in vitro fecal fermentation for 24 h. Total SCFAs refer to the sum of acetic acid, propionic acid, butyrate acid, isobutyric acid, and isovaleric acid. a–e: BBDF (mean ± SD) under different processing conditions with differing letters indicating significant intergroup differences.

The gas production and pH value typically correlated with fermentation, which can reflect the growth of microorganisms in the gut [39]. As shown in Figure 5A, the gas production of all BBDF samples was significantly increased compared with that of the NC group. This result indicates that BBDF can be effectively utilized by gut microorganisms. The enzymatic-extrusion-treated BBDF had significantly higher gas production than the unmodified BBDF, which was mainly attributed to the higher proportion of SDF being more easily utilized by gut microbes, resulting in increased gas production. In addition, ammonia produced by the microbial breakdown of proteins can interact with acidic gases such as carbon dioxide [40].

The fermentation of DF by gut microbiota produces a number of acidic fermentation end products, which in turn affect the pH and microbial composition. As shown in Figure 5B, the enzymatic extrusion BBDF groups had a lower pH than that of the untreated BBDF group, indicating that extrusion and the enzyme mixture improved the ability of BBDF to regulate the pH value. The decrease in the pH value was attributed to the utilization of carbohydrates in the medium by the gut microbes, which led to saccharification and fermentation, followed by the formation of SCFAs, which in turn lowered the pH value [10].

### 3.11. SCFA Analyses

SCFAs are the metabolites of intestinal flora and play an important role in maintaining the function of mucosal immune cells and the integrity of the intestinal epithelium and protecting the intestinal epithelial barrier [39]. The concentration of SCFAs was used as an important index to determine the prebiotic activity of DF. As shown in Figure 5C–H, compared with the blank group, the levels of total SCFAs, acetic acid, propionic acid, butyric acid, and isobutyric acid in the mediums were significantly increased with the addition of BBDFs, and the levels of isovaleric acid were significantly decreased after adding BBDFs. Moreover, the enzymatic extrusion of BBDFs further enlarged the capacity to produce SCFAs. The fermentation of different BBDFs produced the highest acetate yields among the main SCFAs, followed by propionate, butyrate, and isobutyrate. Compared with the unmodified BBDF, the highest concentrations of acetic acid, propionic acid, butyric acid, and isobutyric acid were observed in the L-0.6% group. Each SCFA has its own important role in regulating health. Acetic acid can regulate cholesterol metabolism, enhance insulin secretion, and effectively prevent obesity; propionic acid can inhibit endogenous cholesterol synthesis; butyric acid can act on immune cells through G protein-coupled receptors, which reduces immune cell recruitment and pro-inflammatory signals [9]. The production of acetic and propionic acid in the L-0.6% group was approximately 1.5 times higher than that of the unmodified BBDF, indicating the potential of enzymatic extrusion BBDF for hypoglycemia and hypolipidemia. Moreover, the differences in the contents of butyric and isobutyric acid between the L-0.6% and H-0.6% groups were insignificant, and the contents of both groups were significantly higher than those of the unmodified BBDF group, suggesting that enzymatic extrusion could promote the anti-inflammatory activity of BBDF.

### 3.12. Fecal Microbiota Change

#### 3.12.1. α-Diversity and β-Diversity

**Figure 6:**
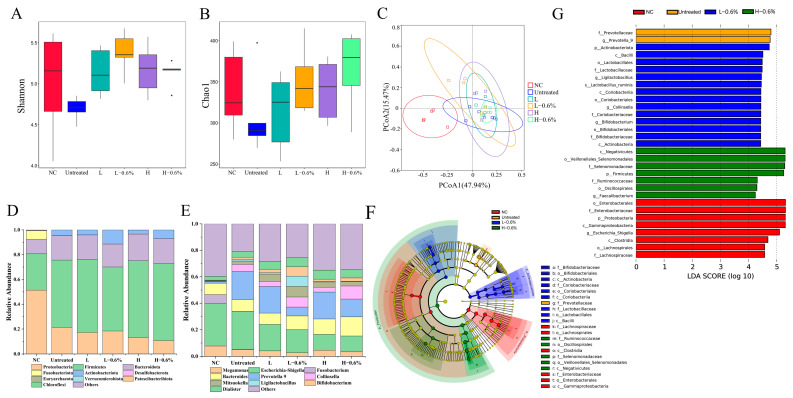
Effects of enzymatic-extrusion-modified BBDF on the gut microbial composition after in vitro fecal fermentation for 24 h. (**A**) Shannon index, (**B**) Chao1 index, (**C**) PCoA analysis, (**D**) gut microbial composition at phylum level, (**E**) gut microbial composition at genus level, (**F**) Cladogram, and (**G**) LEfSe analyses with LDA score.

α-Diversity is commonly expressed in terms of the Shannon index and the Chao1 index, with the Chao1 index indicating colony richness and the Shannon index indicating colony diversity. As shown in Figure 6A,B, the trends in the Shannon index and Chao1 index were approximately the same for all treatment groups. Compared with the NC group, the Chao1 index and Shannon index were decreased in the untreated group but increased in the extrusion and enzymatic extrusion groups. This result indicates that the modification of the physicochemical properties and structural changes in BBDF also alter its fermentation characteristics, which may be related to the changes in the intestinal environment caused by microbial fermentation products [41].

The β-diversity of each group was determined by principal coordinate analysis, as shown in Figure 6C. The microbial community was significantly changed in the NC group after the addition of BBDF, while the microbial community in the sample groups had some similarity. The figure also shows that extrusion changed the microbial community to a certain extent. High-temperature extrusion made the BBDF samples more aggregated and narrowed the confidence intervals. Meanwhile, the dispersion of the samples increased after the addition of the enzyme mixture, and the confidence intervals were widened.

#### 3.12.2. Microbiota Composition Analysis

The predominant phyla and genera of the gut microbiome from the six groups are displayed in Figure 6D,E. A dramatic alteration in the composition of the gut microbiota was observed after fermentation for 24 h. At the phylum level, the colonic microflora in each group was mainly composed of *Bacteroidota*, *Firmicutes*, *Proteobacteria*, and *Actinobacteria*. The relative abundance of *Firmicutes* and *Bacteroidota* increased with the presence of BBDFs compared with the NC group. Proteobacteria in the gut reflect dysbiosis or an unstable gut microbial community structure and can trigger inflammatory responses under certain intestinal environments [21]. The addition of BBDFs inhibited the growth of *Proteobacteria*. Meanwhile, the relative abundance of *Bacteroidota*, *Firmicutes*, and *Actinobacteria* in the group with BBDF significantly increased. Notably, *Fusobacterium*, which was characteristic of the NC group, nearly disappeared after the addition of BBDF. Some studies have shown a significant association between *Fusobacterium* and colorectal cancer, which suggests that BBDF may have an inhibitory effect on colorectal cancer [42].

At the genus level, *Escherichia–Shigella* is known to be detrimental to intestinal health. Its overgrowth leads to intestinal dysbiosis, which promotes the secretion of pro-inflammatory cytokines and induces chronic colitis [9]. After the BBDFs were used as a carbon source, the abundance of *Escherichia–Shigella* decreased compared with the NC group. This result implies that BBDFs have a positive effect on gut health. *Prevotella* 9 is considered to be protective of intestinal health, with immunomodulatory, metabolic modifying, anti-tumor, and intestinal-protecting effects following inflammation in the large intestine [21]. The modified BBDF groups had higher *Prevotella* 9 ratios than that of the control BBDF group, which implies that the modified BBDFs may have an immune-enhancing function. Notably, the relative abundance of *Lactobacillus* and *Bifidobacterium*, which are common probiotics with benefits such as improved immune response and anti-inflammatory properties, was significantly higher in the L-0.6% group.

The microbial community composition was identified by a combination of linear discriminant analysis (LDA) and effect size measurements (LEfSe). As shown in Figure 6F,G, thirty-one species with LDA scores of 4 or higher were statistically different, with eight, two, fourteen, and seven dominant species in the NC, untreated, L-0.6%, and H-0.6% groups, respectively. By contrast, in the LEfSe map species, six, one, nine, and five dominant species were found in the NC, untreated, L-0.6%, and H-0.6% groups, respectively. These combined results showed that the enzymatically extruded BBDF significantly altered the dominant flora of the intestinal microbiota, influenced the microbial degradation pattern, and promoted the colonization of specific microorganisms.

## 4. Conclusions

This study demonstrated that compared to enzyme mixture hydrolysis alone, the dual modification method was more effective in improving the SDF ratio, functional properties, and prebiotic activity of BBDF and decreasing the IDF composition, particle size, viscoelasticity, and structural integrity of BBDF. Under 0.6% (*w*/*w*) enzyme mixture combined with mild extrusion (40–80 °C), the modified BBDF achieved a 3.1-fold increase in the SDF content (10.68% → 32.67%) and exhibited the lowest contents of lignin, cellulose, and hemicellulose, along with the highest capacities for swelling, water holding, oil holding, and glucose and cholesterol adsorption, while achieving the smallest particle size and lowest viscoelasticity. FTIR and XRD experiments indicated that the enzymatic extrusion destroyed the intermolecular interactions and the crystalline regions of the BBDF. These structural changes confirm that mild extrusion conditions can synergize with the enzyme mixture to improve the modification efficiency of DF. Further, the modified BBDFs showed significantly increased gas production, SCFA yields, and microbial diversity during in vitro fecal fermentation, among which the mildly extruded BBDFs exerted superior prebiotic effects. The growth of beneficial bacteria such as *Bacteroidota*, *Firmicutes*, and *Actinobacteria* was fostered in the cultures of modified BBDFs. This encourages further in vivo fermentation tests in animals to provide a comprehensive scientific reference for future studies on enzyme extrusion modification. These findings give insights into the application of enzymatic extrusion in functional BBDF products.

## Figures and Tables

**Table 1:** Effects of enzymatic extrusion on the chemical composition of BBDF (dry basis).

Samples *	Untreated	L	L-0.2%	L-0.6%	H	H-0.2%	H-0.6%
TDF (g/100 g)	79.50 ± 3.28 a	79.21 ± 2.46 a	80.96 ± 1.99 a	78.23 ± 2.23 a	80.32 ± 2.83 a	79.98 ± 2.73 a	80.27 ± 3.11 a
SDF (g/100 g)	10.48 ± 0.14 f	19.94 ± 0.32 e	23.96 ± 0.58 d	32.67 ± 0.77 a	26.22 ± 0.58 c	28.04 ± 0.45 bc	29.44 ± 0.75 b
IDF (g/100 g)	69.02 ± 1.78 a	59.47 ± 1.04 b	57.01 ± 0.99 c	45.56 ± 0.97 f	54.10 ± 1.17 d	51.94 ± 1.75 e	50.83 ± 1.15 e
IDF composition (g/100 g)							
Cellulose	20.12 ± 0.84 a	17.92 ± 1.23 b	11.69 ± 0.70 c	5.41 ± 0.24 e	10.51 ± 0.19 c	7.49 ± 0.07 d	7.20 ± 0.14 d
Hemicellulose	5.61 ± 0.20 b	5.60 ± 0.27 b	4.66 ± 0.22 cd	3.20 ± 0.05 e	5.05 ± 0.25 bc	6.71 ± 0.40 a	4.12 ± 0.12 d
Lignin	42.53 ± 0.22 a	34.67 ± 0.41 c	37.75 ± 1.65 b	30.04 ± 1.39 d	35.69 ± 2.08 bc	34.75 ± 0.16 c	36.83 ± 0.05 bc
Monosaccharide composition (mol %)							
Glc:Ara:Gal:Xyl ^#^	77.85:12.59:5.33:4.23	80.47:10.92:4.86:3.74	80.98:10.48:4.63:3.67	82.82:9.61:4.30:3.27	78.60:11.95:5.14:4.31	78.84:11.91:4.85:4.30	78.91:11.78:4.95:4.36

* L, low-temperature extrusion with a gradient of 40 °C, 50 °C, 60 °C, 70 °C, 80 °C, 70 °C, and 60 °C; H, high-temperature extrusion with a gradient of 80 °C, 90 °C, 100 °C, 110 °C, 120 °C, 110 °C, and 100 °C; 0.2% and 0.6%, the addition ratio (*w*/*w*) of the cellulase–xylanase mixture. ^#^ Ara, arabinose; Gal, galactose; Glc, glucose; Xyl, xylose. a–f: BBDF (mean ± SD) under different processing conditions with differing letters indicating significant intergroup differences.

## Data Availability

The data presented in this study are openly available in Tingting Bu at 10.5281/zenodo.15031701.
